# Environment Enrichment Facilitates Long-Term Memory Consolidation through Behavioral Tagging

**DOI:** 10.1523/ENEURO.0365-22.2023

**Published:** 2023-04-13

**Authors:** Medha Kaushik, Pooja Kaushik, Siddharth Panwar, Shiv Dutt Joshi, Suhel Parvez

**Affiliations:** 1 Department of Toxicology, School of Chemical and Life Sciences, New Delhi 110062, India; 2 School of Computing and Electrical Engineering, Indian Institute of Technology, Mandi, Himachal Pradesh 175075, India; 3 Department of Electrical Engineering, Indian Institute of Technology, Delhi, New Delhi 110016, India

**Keywords:** long-term memory, novel object recognition, novelty exposure, PKMζ, plasticity-related proteins

## Abstract

The behavioral tagging (BT) hypothesis provides crucial insights into the mechanism of long-term memory (LTM) consolidation. Novelty exposure in BT is a decisive step in activating the molecular machinery of memory formation. Several studies have validated BT using different neurobehavioral tasks; however, the novelty given in all studies is open field (OF) exploration. Environment enrichment (EE) is another key experimental paradigm to explore the fundamentals of brain functioning. Recently, several studies have highlighted the importance of EE in enhancing cognition, LTM, and synaptic plasticity. Hence, in the present study, we investigated the effects of different types of novelty on LTM consolidation and plasticity-related protein (PRP) synthesis using the BT phenomenon. Novel object recognition (NOR) was used as the learning task for rodents (male Wistar rats), while OF and EE were two types of novel experiences provided to the rodents. Our results indicated that EE exposure efficiently leads to LTM consolidation through the BT phenomenon. In addition, EE exposure significantly enhances protein kinase Mζ (PKMζ) synthesis in the hippocampus region of the rat brain. However, the OF exposure did not lead to significant PKMζ expression. Further, our results did not find alterations in BDNF expression after EE and OF exposure in the hippocampus. Hence, it is concluded that different types of novelty mediate the BT phenomenon up to the same extent at the behavioral level. However, the implications of different novelties may differ at molecular levels.

## Significance Statement

Given its inherent significance, novelty exposure is frequently prioritized in learning and memory. The behavioral tagging (BT) model for discovering the processes of long-term memory (LTM) consolidation also relies on novelty exploration in its entirety. Yet, the model has not been studied in purview of different types of novelties. Hence, the present study aims to decipher the effects of different novelties, namely, open field exploration and environment enrichment (EE), on the memory consolidation process using the BT phenomenon. Moreover, the implications of novelty-induced behavioral changes on memory-related molecular markers are analyzed. Our findings indicate, for the first time, that EE efficiently consolidates LTM through a BT mechanism, and the process is specific to protein kinase Mζ (PKMζ) synthesis in the hippocampus.

## Introduction

Rooting the phenomenon of memory consolidation through slice electrophysiology, Frey and Morris gave the “synaptic tagging and capture” hypothesis (STC). It was reported that consolidation of late long-term potentiation (L-LTP) from early-LTP (E-LTP) requires the synthesis of specific plasticity-related proteins (PRPs) and their capture by activated synaptic tags (Frey and Morris, 1997, 1998; Parvez et al., 2010). Analogous to the STC study, it was observed in the neurobehavioral studies by Moncada that a weak training of learning task results in the formation of “learning tags.” When such weak training is followed by a novel task such as open field task (OF) within a critical time window, short-term memory (STM) is consolidated into long-term memory (LTM). The phenomenon is termed behavioral tagging (BT). The novel task exposure also triggers PRPs synthesis such as protein kinase Mζ (PKMζ) in the BT model of memory consolidation (Moncada and Viola, 2007; Vishnoi et al., 2016; Naseem et al., 2019).

BT model has been validated using different neurobehavioral paradigms such as spatial object recognition (SOR), inhibitory avoidance task (IAT), contextual fear conditioning (CFC), conditioned taste aversion task (CTA), and novel object recognition (NOR) task, although the novelty provided in the experiments, was same, i.e., OF exploration (Moncada and Viola, 2007; Moncada et al., 2011; Moncada, 2017; Naseem et al., 2019, 2021; Vishnoi et al., 2022). However, there is a paucity of literature providing insights on the effect of different types of novelty on the memory consolidation process and PRPs under the BT hypothesis. Recently, studies have highlighted the potential of environment enrichment (EE) as a non-pharmacological intervention for improving cognitive functions and memory. In addition, long-term EE exposure has been found to restore, modulate, and enhance hippocampal LTP and long-term depression, synaptic plasticity, and learning abilities such as improved food preference for apetitive tasks in rodent models (Artola et al., 2006; Malik and Chattarji, 2012; Morelli et al., 2014; Cintoli et al., 2018; Hullinger et al., 2015; Aghighi Bidgoli et al., 2020). Furthermore, when combined with physical exercise and diet, EE exposure ameliorated spatial memory deficits and restored neurogenesis in the hippocampal region of rats (Kapgal et al., 2016). Thus, it becomes essential to explore the effects of different forms of novelty on the BT process by observing neurobehavior and the potential molecular framework.

PKMζ and the brain-derived neurotrophic factor (BDNF) are two PRPs that are highly regarded for their role in memory consolidation and storage in different brain regions such as the hippocampus, prelimbic cortex, ventral tegmental area and the amygdala (Sacktor, 2008; Katche et al., 2013; M. Kaushik et al., 2022). PKMζ was first identified as an essential molecular player for the consolidation of E-LTP into L-LTP. Further, it was observed that PKMζ actively participates in AMPA receptor (AMPAR) trafficking at synapses, thus enhancing the synaptic strength (Sacktor et al., 1993; Sacktor, 2008; Sacktor and Fenton, 2018). Similarly, BDNF has been observed for its role in local protein synthesis and regulation of cytoskeletal dynamics in dendritic spines, producing synaptic strength and L-LTP in the hippocampus. Interestingly, BDNF also sustains L-LTP through PKMζ, establishing a crosslink between two memory- related markers (Mei et al., 2011; Panja and Bramham, 2014). PKMζ and BDNF have been established as the PRP candidates involved in consolidating LTM in the prelimbic cortex through the BT paradigm (Naseem et al., 2019, 2021).

Thus, the present study aimed to explore the effect of different novelties (OF and EE) on the BT phenomenon by using NOR as the learning task for rodents. In addition, memory- related molecular framework and plasticity changes were analyzed using appropriate molecular techniques. Overall, the current work suggests the modulatory role of different forms of novelty in the memory consolidation process through neurobehavior and PRP expressions.

## Materials and Methods

### Ethics

All experiments were performed in accordance with standard guidelines of the Institutional Animal Ethics Committee (IAEC). The IAEC is recognized by the Committee for the Purpose of Control and Supervision of Experiments on Animals (CPCSEA). Ethical approval for the present study was taken from IAEC, followed by procurement of animals from Central Animal House Facility (CAHF) of the institute.

### Experimental design

**Figure 1.**
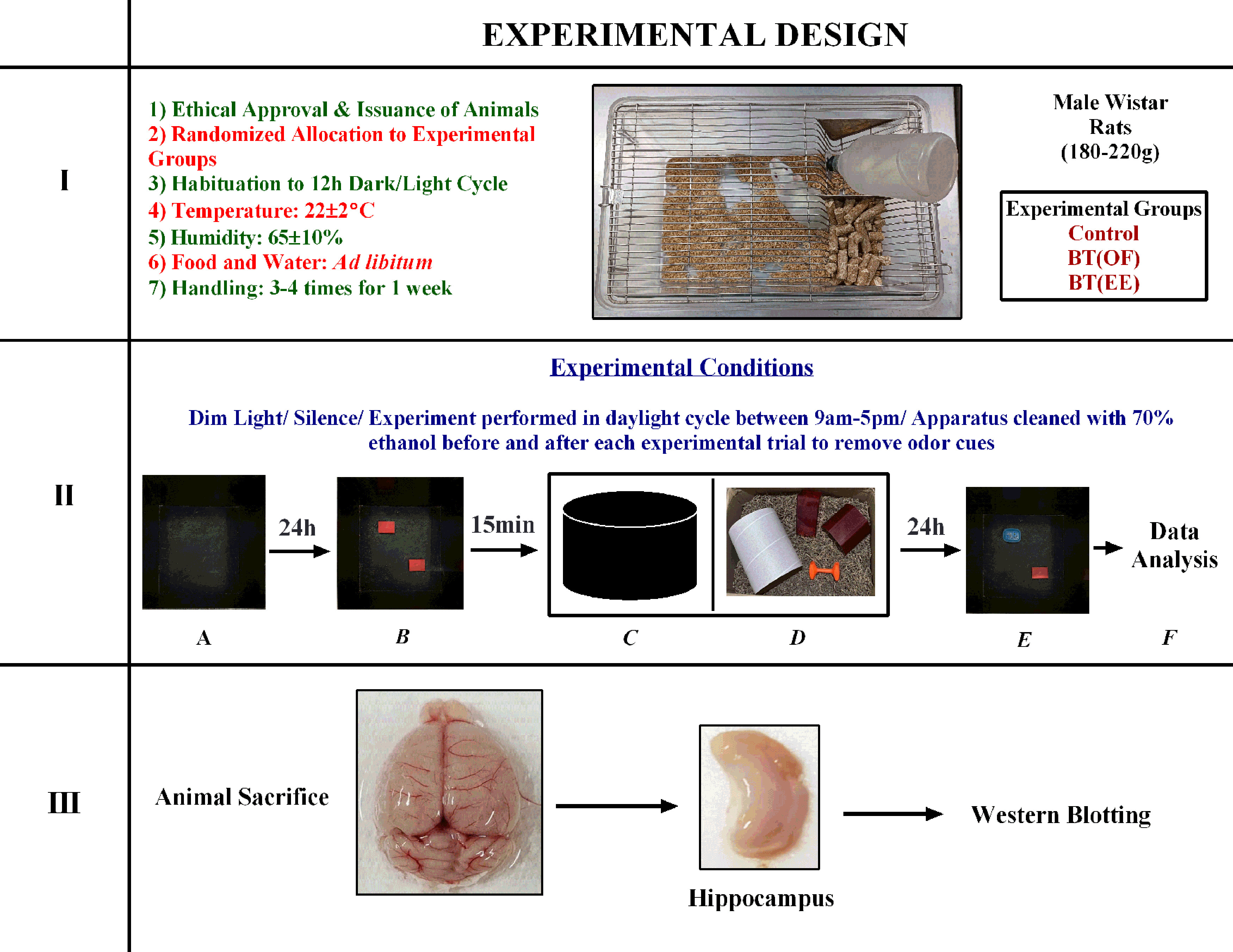
Schematic representation of experimental design. **I**, Ethical approval for animal experimentation was taken from IAEC, followed by procurement of adult male Wistar rats (180–220 g) from CAHF. Rats were randomly allocated to three experimental groups and maintained at standard housing conditions. All animals were handled for three to four times for one week before starting the experiment for the purpose of familiarization. **II**, Experimental conditions such as dim light, silence, cleanliness were maintained on all behavioral experiment days. All experiments were performed during the daylight cycle of animals between 9 A.M. to 5 P.M. NOR apparatus was cleaned with 70% ethanol before and after each animal trial to remove the odor cues. ***A***, Habituation. Animals were habituated with empty NOR apparatus for 5 min on day 1. ***B***, Training. Two similar shaped objects were placed diagonally in the NOR apparatus and animals were given weak training (single trial) for 5 min. ***C***, BT(OF) group rats were further exposed to 5-min open field exploration after 15 min of NOR training. ***D***, BT(EE) group rats were given 5-min enriched environment exposure after 15 min of NOR training. The enriched environment comprised of an extralarge fun tunnel for rats, a hut, nesting material such as sizzle pet and dumbbell. ***E***, Test. One object was replaced with a novel object with different shape and color in the NOR apparatus. Animals were tested for LTM 24 h after training session. ***F***, NOR behavioral data were analyzed for percentage of novel object exploration, object exploration time and number of entries in the novel zone. **III**, After test session, animal brain was excised and hippocampus was snap frozen for analyzing protein expression changes through western blotting.

Total 33 adult male Wistar rats (age range from eight to nine weeks; weight range from 180 to 220 g) were used for the study (Naseem et al., 2021). All animals were housed at CAHF in polycarbonate cages with dry rice husk as the bedding material. Two rats per cage were housed and all animals were subjected to 12/12 h light/dark cycle with food and water *ad libitum*. Housing temperature was maintained at 22 ± 2°C with relative humidity of 65 ± 10%. Rats were randomly divided into three groups: control, behavioral tagging induced with open field [BT(OF)], and behavioral tagging induced with environmental enrichment [BT(EE)]. For one week, all rats were handled by the experimenter for 2–3 min of daily for the purpose of familiarization. Afterwards, rats were subjected to NOR task, OF task and EE, as described in Table 1. A schematic representation of experimental design is given in Figure 1.

**Table 1:** Detailed experimental protocol for neurobehavioral analysis

	Control (*n* = 11)	BT(OF) (*n* = 11)	BT(EE) (*n* = 11)
Day 1: habituation	Rats were placed in an empty NOR apparatus and were allowed to freely explore the arena for 5 min.		
Day 2: training	Two similar shaped objects were placed in NOR apparatus and rats were allowed to explore the objects for 5 min. Single trial training was provided to all groups.		
	No novelty exposure was provided	15 min after training, rats were subjected to 5-min open field task as novelty	15 min after training, rats were subjected to 5-min environment enrichment as novelty
Day 3: test	One object was replaced with a novel object in NOR apparatus and rats were tested for 5 min for NOR long-term memory		

Experimental protocol for studying the effect of different types of novelty exposure on memory consolidation process. Animals were randomly divided into three experimental groups: control, BT(OF), and BT(EE) with *n* = 11 for each group. All groups were subjected to NOR as the learning task, while OF and EE were two different types of novelties used to induce the BT phenomenon. Control group was not given novelty exposure. Complete neurobehavioral experiment consisted of three days protocol following 5 min of habituation, single trial training (followed by novelty for two groups) and testing session.

### Novel object recognition task

NOR is a well-established neurobehavioral task for evaluation of the recognition memory in rodents. The task was performed according to the method described previously (Vishnoi et al., 2022) with slight modifications. The NOR apparatus was a rectangular chamber with dimensions (50 × 50 × 35 cm). The apparatus was well equipped with a digital camera placed at the top of the chamber providing the overhead view. All neurobehavioral observations were recorded using ANY-maze software v6.2 (Stoelting Co.) connected to the apparatus. On the first day, rats were habituated with the apparatus for 5 min without any objects placed in the chamber. After 24 h, during training session, two similar shaped rectangular objects were placed in the apparatus diagonally. All rats were subjected to single trial training of NOR for 5 min. For the test session, one object was replaced with a novel object with different shape and color and rats were tested for object recognition LTM for 5 min, 24 h after training.

### Open field task

OF is readily used for assessing the locomotor and exploratory activity of rodents. However, in the present study, OF task was used as the novel experience after NOR task. After 15 min of NOR training, animals of only BT(OF) group were subjected to open field exploration. The OF apparatus was a circular empty tank with black surface and walls. Rats were placed at the center of the apparatus and were allowed to freely explore the arena for 5 min. Animals of control and BT(EE) group were not subjected to open field task (Naseem et al., 2021; Vishnoi et al., 2022).

### Environment enrichment

EE has been explored as a recreational task for rodents to analyze the improvement in their health in a diseased/stressed state. Hence, EE was another type of novel experience used in the present study after NOR task. Animals of only BT(EE) group were given enriched environment experience 15 min after NOR training task. The enriched environment consisted of several toys and objects for providing a playful and enriched environment for animals. Sizzle pet, a type of nesting material, a hut for rat, small dumbbells for playful activity, extralarge fun tunnel and rat retreat chamber (LBS Biotechnology, United Kingdom) were used as the EE objects. All EE objects were placed inside the OF apparatus and rats were allowed to freely explore the EE set-up for 5 min. Animals of control and BT(OF) group were not subjected to EE task (De Virgiliis et al., 2020).

### Western blotting

On the test day, rats were immediately sacrificed after the NOR test for excision of brain. Hippocampal tissue from both the hemispheres of brain was collected, snap frozen and stored at −80°C for western blot analysis. Briefly, tissue homogenate was prepared in lysis buffer (20 mm Tris–HCl with pH 7.5; 150 mm NaCl, 2 mm EDTA; 0.1% NP-40, 0.1% SDS, and 1× protease inhibitor cocktail). The homogenized sample was sonicated and centrifuged. Finally, supernatant was collected and protein quantification was done using Bradford assay. Samples were resolved using 10–12% SDS-PAGE gel at 20 mA, followed by transfer to PVDF membranes for 1.5–2 h at 150 mA using Mini Trans-Blot Cell apparatus (Bio-Rad). Afterwards, membranes were blocked using 5% non-fat skim milk (Sigma-Aldrich) at room temperature for 1 h followed by 3× PBST washing of 5 min each. The membranes were then immunoblotted with PKMζ antibody (1:1000, Abcam, catalog #ab59364) and BDNF antibody (1:1000, Genetex, catalog #GTX132621) overnight at 4°C. Next day, 3× PBST washing was done and membranes were incubated with horseradish peroxidase-conjugated anti-rabbit secondary antibody (1:10,000, Invitrogen). Enhanced chemi-luminescence reaction substrate (ECL; Thermo Scientific) was used for the visualization of bands followed by densitometric analysis using ImageJ software (NIH; P. Kaushik et al., 2021; Naseem et al., 2021).

### Statistical analysis

All data were analyzed using GraphPad Prism 6 software (GraphPad Software Inc.) and represented as mean ± SEM. ANOVA followed by Tukey’s test was applied for comparison of three experimental groups. Mann–Whitney *U* test was used to compare two independent groups. Values of *p *<* *0.05 were considered statistically significant.

## Results

### EE-mediated novelty induces NOR-LTM through BT phenomenon

**Figure 2.**
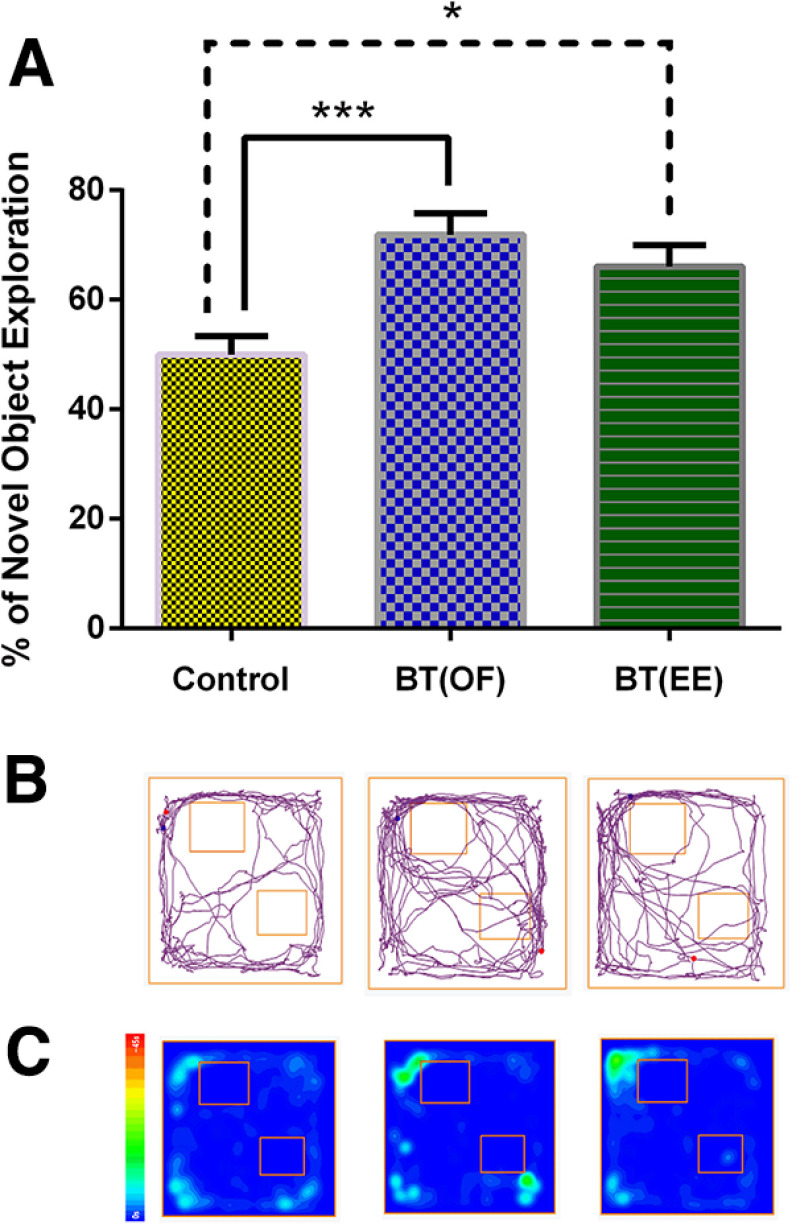
Percentage of novel object exploration was calculated for all experimental groups to study the neurobehavioral effects of different types of novelties, namely, OF and EE, on LTM consolidation through BT phenomenon. ***A***, Statistically significant differences in the percentage of novel object exploration were seen after OF and EE exposure when compared with control group (***p < 0.001 and *p < 0.05, one way ANOVA, *n* = 11, data represented as mean ± SEM) when tested 24 h after training session. ***B***, Exploratory track plots of control, BT(OF), and BT(EE) group during 5-min NOR test session generated from ANY-maze software. ***C***, Exploratory heat maps of control, BT(OF), and BT(EE) group during test session generated from ANY-maze software. The data are suggestive of EE modulated BT with OF as a positive control. Different type of novelty produces similar extent of LTM consolidation via BT phenomenon when compared with control group in which no novelty was provided.

Research has established that 5 min OF exploration (novelty) within a critical time window after single trial training (weak training) efficiently consolidates STM to LTM in NOR task through BT process (Naseem et al., 2019, 2021; Vishnoi et al., 2022). Additionally, BT has been established through IAT, SOR, CFC, and NOR as the learning events, while OF exploration was used as the novelty in all such studies (Moncada and Viola, 2007; Moncada et al., 2011; Moncada, 2017; Naseem et al., 2019, 2021; Vishnoi et al., 2022). However, to the best of our knowledge, experiments have not been done to explore the effect of different novelties in memory consolidation through BT paradigm by any research group. Thus, the primary objective of the present study was to investigate whether a different type of novelty enhances the process of memory consolidation. EE has been well studied in rodents for its modulatory effects on behavior, cognition, and memory. Hence, we investigated the role of EE as a novelty in consolidation of NOR-LTM through BT mechanism. OF exploration as a novelty was again validated in the present study as a positive control. For the first time, our results found that even 5 min of EE exposure after single trial NOR training results in LTM consolidation when tested 24 h after NOR training session. Our results depict significant increase in the percentage of novel object exploration of both BT(EE) and BT(OF) group when compared with control group (*F*_(2,30)_ = 9.191; ***p < 0.001, *p < 0.05, ordinary one-way ANOVA followed by Tukey’s test, *n* = 11; Fig. 2*A*). The exploratory behavior track plots and heat map of animals also support the neurobehavioral analysis (Fig. 2*B*,*C*). It is thus inferred that even 5 min of EE as a novelty exploration after single trial weak training induces LTM via BT mechanism.

**Figure 3.**
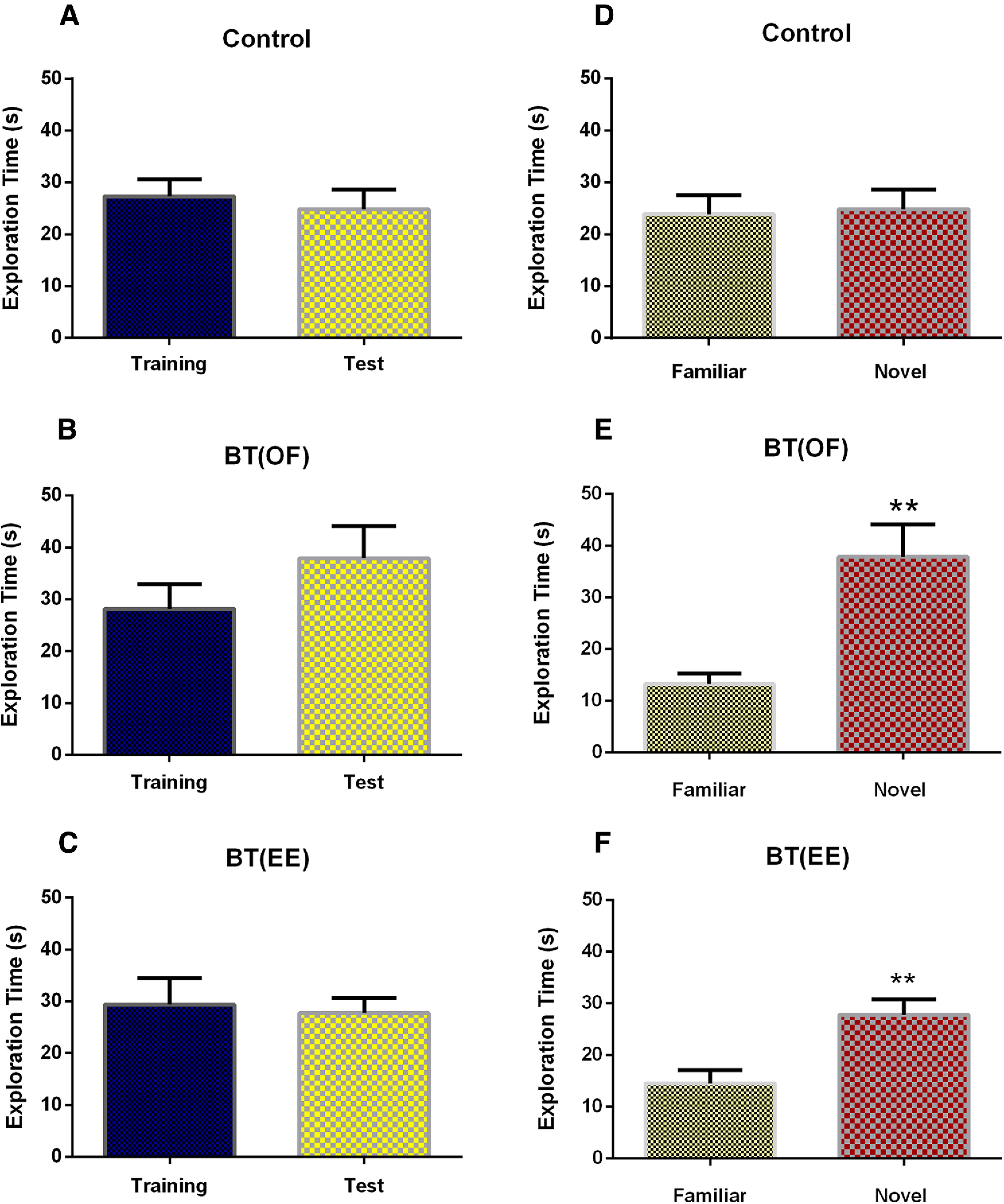
Intragroup comparison of object exploration time for control, BT(OF), and BT(EE) groups. ***A–C***, Object exploration time in training versus test session represented as mean ± SEM. No significant difference was observed (*p* > 0.05, Mann–Whitney *U* test, *n* = 11) in any experimental set, when compared within the groups. The object which was replaced with a novel object on test day was considered as the object of interest for calculating the exploration time for training session, while exploration time of novel object was used for the test session. ***D–F***, Familiar versus novel object exploration time during test session represented as mean ± SEM. Significant differences were observed in novel object exploration time for BT(OF) and BT(EE) groups when intragroup comparison was done with familiar object exploration time (**p < 0.01, Mann–Whitney *U* test, *n* = 11) during 5-min NOR test session. The data suggest increased novel object exploration after OF and EE exposure, indicative of LTM formation.

Figure 3*A–C* shows within the group comparison of object exploration time between training and test session with nonsignificant differences [control: *U *=* *52.0, *p* = 0.595, Mann–Whitney *U* test, *n* = 11; BT(OF): *U *=* *44.0, *p* = 0.292, Mann–Whitney *U* test, *n* = 11; BT(EE): *U *=* *57.0 *p* = 0.831, Mann–Whitney *U* test, *n* = 11]. In Figure 3*D*, control rats (without novelty exposure) almost equally explored the familiar and novel object thus reflecting non-significant difference in the exploration time (*U *=* *55.5, *p* = 0.759, Mann–Whitney *U* test, *n* = 11). BT(OF) rats spent significant time in exploring the novel object as compared with the familiar object (*U *=* *14.0, **p < 0.01, Mann–Whitney *U* test, *n* = 11; Fig. 3*E*). Similarly, BT(EE) rats significantly explored novel object as compared with the familiar object during test session (*U *=* *20.5, **p < 0.01, Mann–Whitney *U* test, *n* = 11; Fig. 3*F*). The within the group comparison of exploration time for familiar versus novel object supports our previous findings that EE induces NOR-LTM consolidation through BT phenomenon.

**Figure 4.**
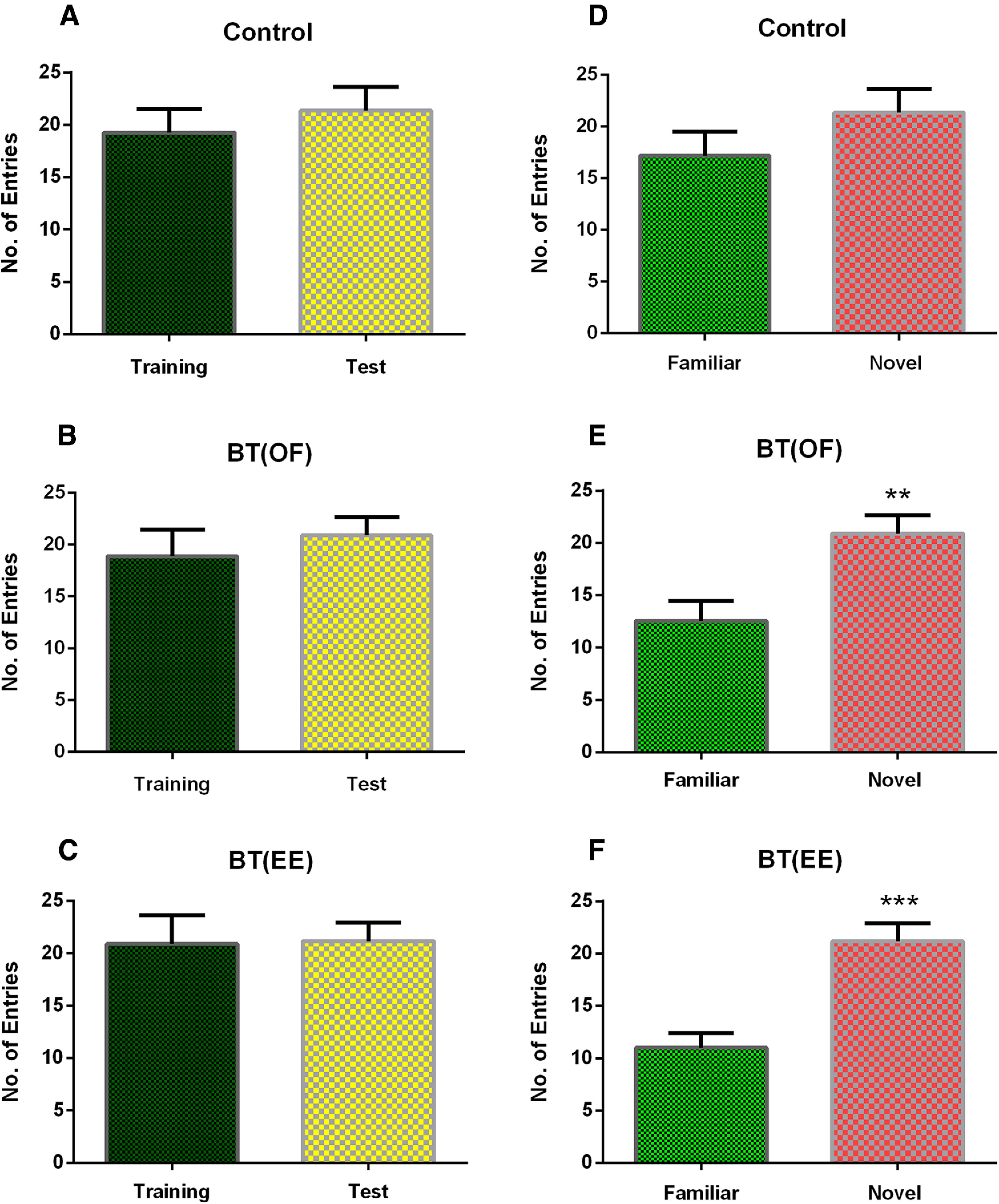
Results depict intragroup comparison of number of entries for control, BT(OF), and BT(EE) groups. ***A–C***, No significant difference was observed (*p* > 0.05, Mann–Whitney *U* test, *n* = 11, data represented as mean ± SEM) in any experimental set for number of entries in training versus test session. The zone in which novel object was replaced from familiar object was used as the zone of interest for training session, while the novel object zone was considered for test session. ***D–F***, Number of entries in familiar and novel zone during test session represented as mean ± SEM. Significant differences were observed in novel zone entries for BT(OF) and BT(EE) groups when compared with familiar zone entries (**p < 0.01 and ***p < 0.001, Mann–Whitney *U* test, *n* = 11) during 5-min NOR test session. The data are again indicative of LTM consolidation process with increased entries to novel zone after novelty exposure when compared within the group as well.

Analysis of number of entries to the novel and familiar zones also highlights the effect of novelty exploration on memory consolidation process. When compared between training and test data for all three groups, no significant difference was found in the number of entries to the desired zone within the individual groups [control: *U *=* *48.0, *p* = 0.427, Mann–Whitney *U* test, *n* = 11; BT(OF): *U *=* *52.0, *p* = 0.594, Mann–Whitney *U* test, *n* = 11; BT(EE): *U *= 54.5, *p* = 0.710, Mann–Whitney *U* test, *n* = 11; Fig. 4*A–C*]. However, during test session, entries to novel zone significantly improved for BT(OF) (*U *=* *19.0, **p < 0.01, Mann–Whitney *U* test, *n* = 11; Fig. 4*E*) and BT(EE) (*U *= 9.5, ***p < 0.001, Mann–Whitney test, *n* = 11; Fig. 4*F*) groups, while control group depicted non-significant difference in the entries to novel zone (*U *=* *38.0, *p* = 0.145, Mann–Whitney *U* test, *n* = 11; Fig. 4*D*). Thus, taken together it has been found that EE as a novelty significantly contributes to the memory consolidation process in the same way as the OF novelty through BT mechanism.

### EE exposure induces PKMζ synthesis but not BDNF

**Figure 5.**
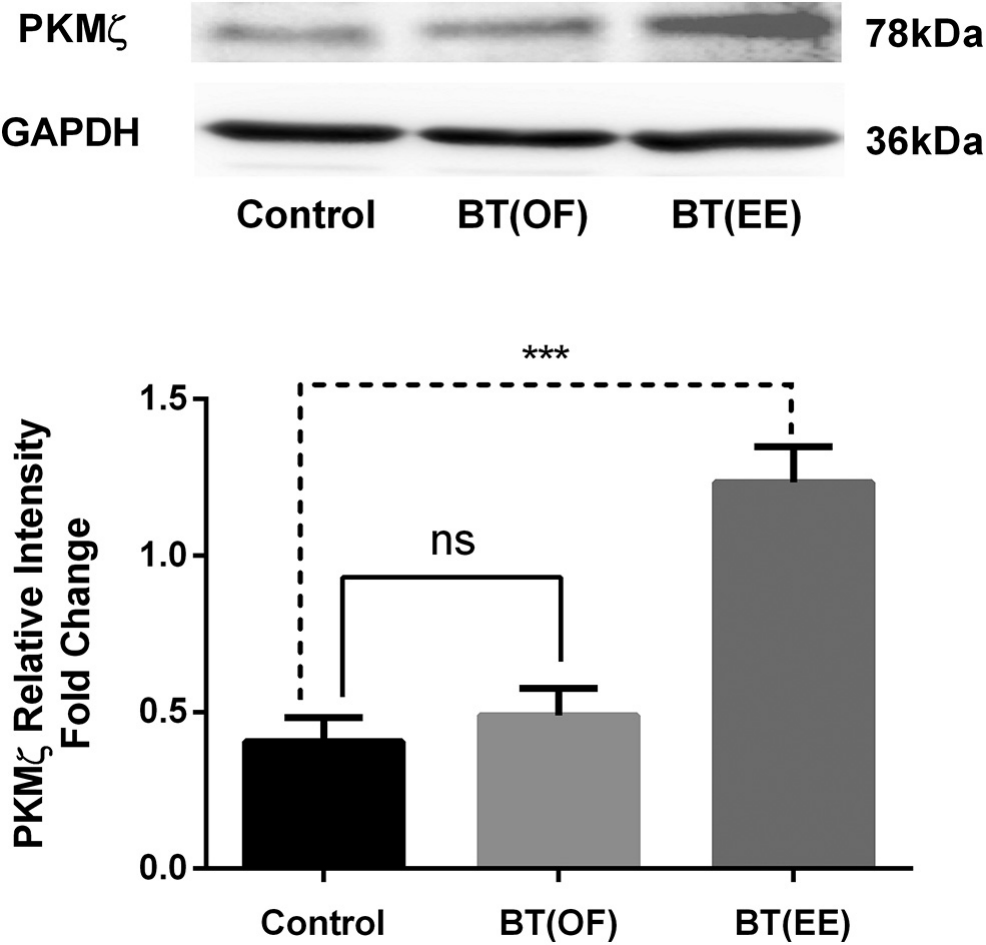
Plasticity related molecular markers induced by different forms of novelty through BT phenomenon was observed. Expression of PKMζ after OF and EE exploration was analyzed using western blotting in hippocampal tissue. GAPDH was used as the normalizing factor for PKMζ relative intensity fold change. Significantly enhanced levels of PKMζ protein in hippocampus were observed in BT(EE) group (***p < 0.001, one way ANOVA) when compared with control. However, no significant difference was found in the PKMζ expression for BT(OF) group when compared with control (ns, non-significant; one-way ANOVA). All data represented as mean ± SEM (*n* = 4). The data suggest strong induction of PKMζ synthesis in hippocampus of rat brain after EE exposure, indicating the differences in molecular alterations by different form of novelties. The synthesis of PKMζ after EE exploration supports the neurobehavioral findings and establishes EE as the better form of novelty as compared with OF for hippocampus dependent learnings. For full blot image of PKMζ, see Extended Data Figure 5-1; for full blot image of GAPDH, see Extended Data Figure 5-2.

Previous studies have established that novelty exposure after weak stimulus/training results in the synthesis PRPs (Parvez et al., 2010; Sacktor and Fenton, 2018; Naseem et al., 2019). Hence, in this study we also aimed to investigate the effect of different novelties (OF and EE) on the expression of PRPs in the hippocampus of rat brain. *In vitro* studies on rat hippocampal slices have found the role of PKMζ in the maintenance of L-LTP (Sacktor and Fenton, 2018; Awasthi et al., 2019). Literature has also established PKMζ as the LTM specific PRP in the prelimbic cortex region of brain after BT (Naseem et al., 2019). However, there are evidences of effect of EE on hippocampal molecular signatures (Zanca et al., 2015; Chen et al., 2016). Thus, initially, we examined the PKMζ expression through western blotting in the rat hippocampal tissue after exposure to OF and EE as novelties. Interestingly, it was found that PKMζ was significantly expressed in the hippocampus of the BT(EE) group when compared with control. At the same time, the expression was non-significant for BT(OF) versus control group (*F*_(2,9)_ = 23.69, ***p < 0.001, ordinary one-way ANOVA, *n* = 4; Fig. 5).

10.1523/ENEURO.0365-22.2023.f5-1 Extended Data Figure 5-1 Full blot image of PKMζ. Download Figure 5-1, TIF file.

10.1523/ENEURO.0365-22.2023.f5-2 Extended Data Figure 5-2 Full blot image of GAPDH. Download Figure 5-2, TIF file.

**Figure 6.**
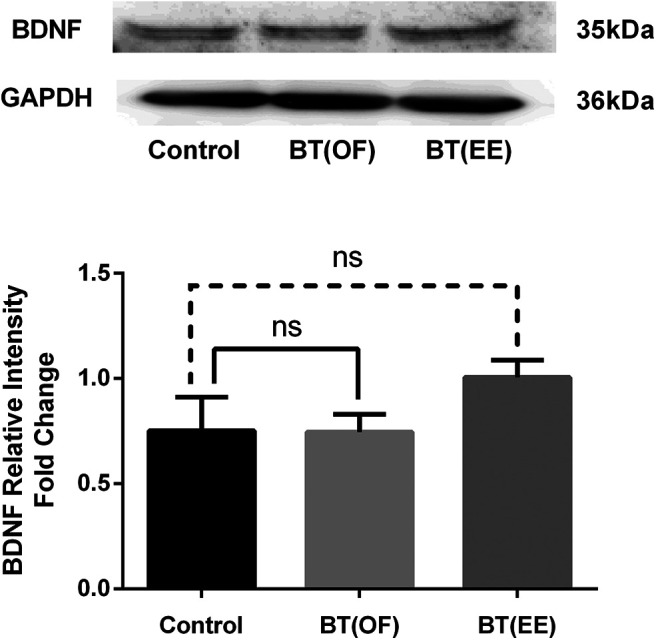
Expression of BDNF in hippocampus after OF and EE as different novelty explorations was observed. GAPDH was used as the normalizing factor for BDNF relative intensity fold change. No significant differences were found in the expression of BDNF in hippocampus region after OF and EE exploration when compared with control group (ns, non-significant; one-way ANOVA). All data represented as mean ± SEM (*n* = 4). For full blot image of BDNF, see Extended Data Figure 6-1.

Similarly, BDNF is another molecular marker widely studied for its role in the memory consolidation. Research has also established the role of BDNF in LTM consolidation through BT in the prelimbic cortex of the rat brain (Naseem et al., 2021). Furthermore, studies established the functionalities of BDNF after EE exposure in the the hippocampus region (Wang et al., 2019; Xu et al., 2021). Hence, we investigated the effect of EE and OF exploration on BDNF expression in the hippocampus region of the rat brain. However, our results depict a non-significant increasing trend of BDNF expression in the BT(EE) group compared with the control group. In contrast, the BT(OF) group observed an almost similar expression of BDNF as that of the control group (*F*_(2,9)_ = 1.678, *p* = 0.2402, one-way ANOVA, *n* = 4; Fig. 6).

10.1523/ENEURO.0365-22.2023.f6-1 Extended Data Figure 6-1 Full blot image of BDNF. Download Figure 6-1, TIF file.

Thus, the present molecular findings reflect EE’s effect on a molecular level, resulting in enhanced synthesis of PKMζ but not BDNF. On the other hand, OF exploration does exhibit LTM consolidation, but the changes at the molecular level were not evident in the hippocampus region from the present study.

## Discussion

Animal research related to the effects of novelty on learning and memory is prolific. However, there is a shortage of conclusive findings regarding the different aspects of novelty that exert strong neuronal stimulations and activate the memory consolidation cascade. Although novelty exposure forms the backbone of the BT hypothesis, limited studies have explored different novelties for LTM consolidation in the BT model. Our behavioral findings for the first time suggest that apart from OF, even a short-term EE exposure (5 min) as a novelty within a critical time window (within 1–1.5 h after a weak NOR learning task) significantly improves memory consolidation in adult rats by BT mechanism. Moreover, the effect of EE novelty was similar to that of the OF novelty when compared with control rats. The behavioral findings are parallel with the previous studies portraying the BT phenomenon for the memory consolidation using the OF paradigm as the novel exposure (Moncada, 2017; Naseem et al., 2021; Vishnoi et al., 2022). Additionally, our findings corroborated with existing evidence that EE helps in the enhancement of cognition and memory through several cellular and molecular cascades (Hullinger et al., 2015; Cintoli et al., 2018; Stazi et al., 2023).

The implication of behavioral experiences on molecular signatures provide robust proof of a phenomenon. Similarly, behavioral findings of different forms of novelty exposure in the BT model also require validation of memory-related molecular expressions. As reported, novelty exposure in BT initiates the synthesis of PRPs, which pairs with learning tags formed because of weak training, thus facilitating LTM consolidation. As a result, PKMζ is one such LTM-specific PRP that is actively synthesized for the memory consolidation (Helfer and Shultz, 2018). Our results found significantly increased levels of PKMζ in the hippocampus of EE-exposed rats compared with the control group. These results strongly indicate that even a short-term exposure (5 min) of EE as a novelty efficiently triggered the synthesis of LTM-specific PRP through the BT mechanism. Our findings are consistent with previous studies as well. It has been found that EE exposure enhances PKMζ synthesis in the hippocampus of rats by increasing the glucocorticoid receptors, thus reducing PKMζ trafficking. In addition, EE reverses the hypermethylation of PKMζ, resulting in the enhancement of cognitive functions in the prelimbic cortex (Zanca et al., 2015; Chen et al., 2016; Yan et al., 2018). Since tissue was collected immediately after the test, there could be a possibility of higher expression of PRPs because of behavior at test. However, this was clear with the interpretation of molecular data in which protein expression was non-significant in the control group, highlighting the novelty exposure specific synthesis of PKMζ. Similar findings have been reported by previous studies as well (Naseem et al., 2019, 2021).

On the other hand, previously, PKMζ synthesis and expression has been reported in the BT(OF) group within the prelimbic cortex region (Naseem et al., 2019). However, the same was not observed in the hippocampus region in the present study. It may be that the expression of PKMζ in the hippocampus may require a much stronger novel event than the OF. Thus, based on present molecular findings EE has been anticipated as a better form of novelty compared with OF for inducing BT. Supplementary studies are required to study the effect of stronger novel exposures on hippocampal PKMζ synthesis compared with OF and EE to rule out the confounding factors.

Similarly, BDNF/TrkB pathway has also been activated after EE exposure in rodents, improving their LTM impairment and aberrant synaptic plasticity (Giacobbo et al., 2019; Wang et al., 2019; Santoso et al., 2020; Xu et al., 2021). However, our findings suggest a non-significant increasing trend of BDNF expression in the hippocampus region after EE exposure compared with the control group. This observation was similar to the findings of, where short-term EE exposure enhanced hippocampal synaptic plasticity, yet, BDNF alteration was not observed (Stein et al., 2016). Another study also provides similar evidence of EE facilitated hippocampal synaptic plasticity but lower expression of Ras activity, which is a downstream cascade of BDNF signaling (Novkovic et al., 2015). It is argued that the lack of alterations in hippocampal BDNF levels may be because of individual rat variability and may require a much stronger novelty exposure. Also, there is a lack of literature to show that BDNF expression is necessary for the memory modifications induced by EE. It must be noted that the age, timing, and length of exposure to EE all affect the reported increase in molecular expression levels of BDNF. Because of the significant differences in experimental conditions among the animals, the studies show a higher risk of biasedness (Barros et al., 2019).

**Figure 7.**
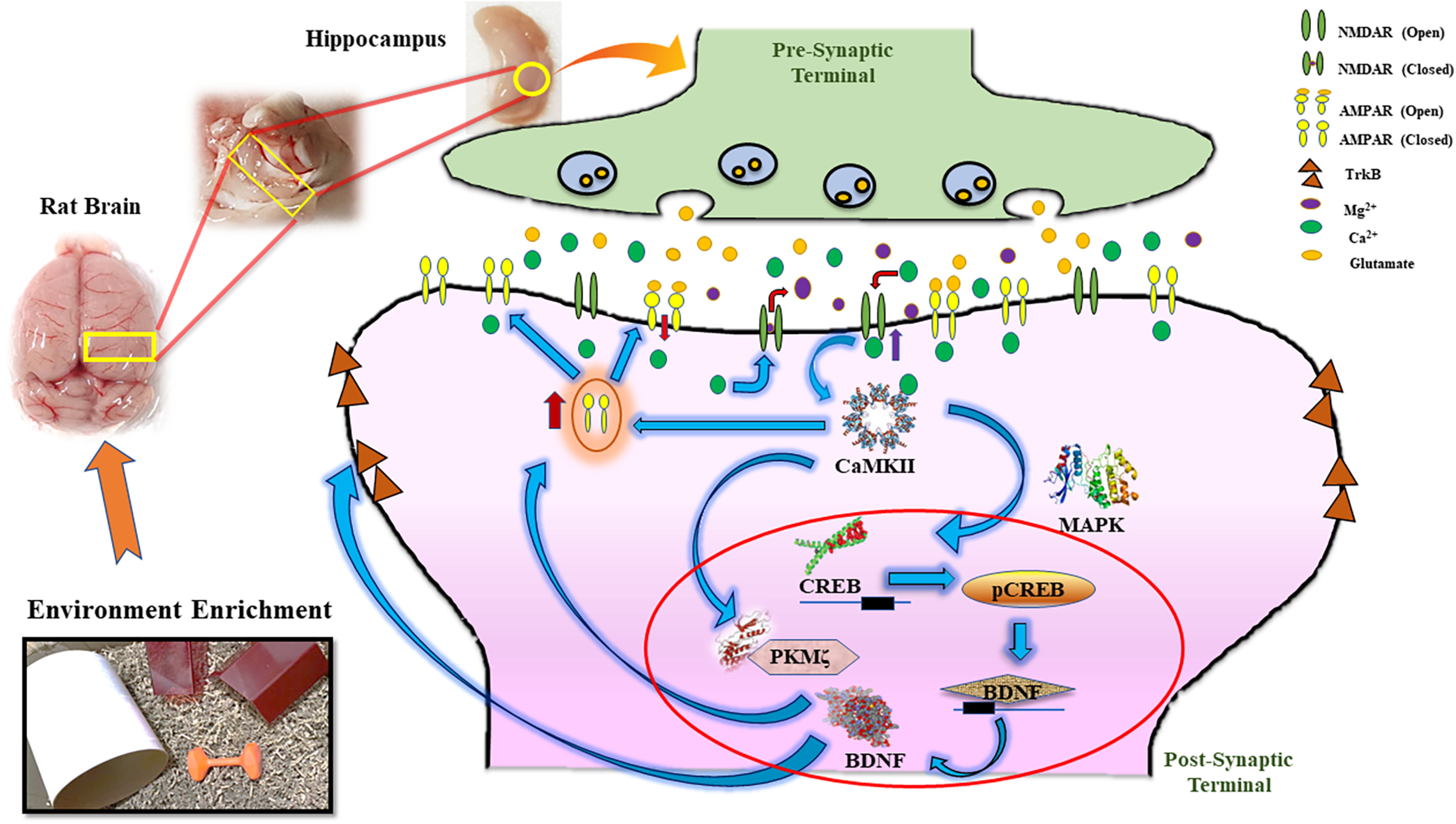
Schematic representation of molecular cascade activated in the hippocampus of rat brain after EE exposure as a process of LTM consolidation through BT phenomenon. Exposure to EE as a novelty after weak training stimulates the release of stored glutamate into the synaptic cleft from presynaptic terminal. The released glutamate is then captured by α-amino-3-hydroxy-5-methyl-4-isoxazolepropionic acid (AMPA) receptors on the post-synaptic terminal, resulting in the opening of AMPA receptors and increased influx of Na^+^ ions in the dendritic space. The increased levels of influxed Na^+^ ions further help in activating the NMDA receptors by forcing the Mg^2+^ into the synaptic space. NMDA receptor activation ensures higher influx of Ca^2+^ into the dendritic space, which binds to the calcium/calmodulin kinase II receptors (CaMKII), thus activating the several kinases such as MAPK inside the cell. In addition, CaMKII mediates the exocytosis of AMPA receptors on the postsynaptic surface, thus further enhancing the synaptic strength and increased activation of synapse for neuronal signaling. Moreover, PKMζ synthesis and CREB phosphorylation are initiated with stronger stimulation of CaMKII by virtue of novelty exposure. Phosphorylated CREB further upregulates the transcription of BDNF protein which in turn enhances the AMPA receptor trafficking as well as activation of its receptor TrkB. The cascade thus facilitates LTM consolidation process after stimulation from EE exposure as the novelty establishing BT as a molecular phenomenon for memory formation.

Finally, the present findings provide first hand evidence for the EE modulated BT for LTM consolidation following the NOR task. Our results found that even 5-min EE exposure as a novelty is sufficient for synthesizing and expressing LTM-specific PRP PKMζ in the hippocampus region. Based on molecular findings, it is concluded that EE as a novelty exposure follows the PKMζ-specific trajectory in the hippocampus region for facilitating the LTM consolidation through the BT phenomenon. Nevertheless, further studies are required to explore the phenomenon of BDNF signaling in the hippocampus after exposure to different novelty forms. Since BDNF/TrkB and BDNF-CREB signaling are a well-established phenomenon in the memory consolidation process and studies have also observed EE exposure with enhanced BDNF expressions in the hippocampus (Giacobbo et al., 2019; Wang et al., 2019; Santoso et al., 2020; Xu et al., 2021), it is a possibility that CaMKII-BDNF-CREB signaling machinery is not activated because of unknown reasons up to its full potential in BT model (Fig. 7). Future work intends to highlight the aspects of CaMKII-BDNF-CREB signaling after different novel exposures in the BT model of learning and memory. In addition, neurobehavioral studies are highly susceptible to alterations because of several uncontrollable external factors, rigor, and reproducibility of behavioral data. All of this makes interpreting data at the molecular level a huge challenge. However, such behavioral susceptibility is a necessity as well to unfold the molecular mysteries of learning and memory in its entirety. Thus, it is argued that molecular implications of behavioral data are not always appropriately translated, producing confounding results in many cases. Thus, additional studies are warranted under different conditions.

## Synthesis

Reviewing Editor: Jonathan Lee, University of Birmingham Decisions are customarily a result of the Reviewing Editor and the peer reviewers coming together and discussing their recommendations until a consensus is reached. When revisions are invited, a fact-based synthesis statement explaining their decision and outlining what is needed to prepare a revision will be listed below. The following reviewer(s) agreed to reveal their identity: Peter Serrano.

The authors’ considered response to our previous synthesis of reviews is very welcome. Our concerns have been mostly addressed in the response. While readers might have benefitted from the insight afforded by the response, there are understandable space constraints.

One outstanding issue remains the timing of tissue collection. As we continue to understand it, the brains are taken immediately after the final test. Yet the interpretation is linked to the prior EE (vs OF) experience. Therefore, the levels of PKMz (and BDNF) are somewhat confounded with the behavioral differences at the test. Ultimately, this probably does not change the overall interpretation of the data, but it would be good and transparent to acknowledge this in the discussion; e.g. to what extent could the protein levels have been affected by the test? Presumably, the ideal woud have been to take the brains from a separate set of rats that went through the behavioral tagging procedure, but were killed after OF/EE instead of being tested. However, your approach has reduced the number of rats used.
